# Meta-analyses in paediatric surgery are often fragile: implications and consequences

**DOI:** 10.1007/s00383-020-04827-5

**Published:** 2021-01-16

**Authors:** Arne Schröder, Oliver J. Muensterer, Christina Oetzmann von Sochaczewski

**Affiliations:** 1 Klinik für Kinder- und Jugendmedizin, Elisabeth-Krankenhaus, Essen, Germany; 2 Klinik und Poliklinik für Kinderchirurgie, Universitätsmedizin der Johannes-Gutenberg-Universität, Langenbeckstraße 1, 55131 Mainz, Germany; 3 Kinderchirurgische Klinik und Poliklinik, Dr. von Haunersches Kinderspital, Ludwig-Maximilians-Universität, München, Germany; 4 Klinik für Kinder- und Jugendmedizin, Klinikum Dortmund, Dortmund, Germany; 5 Sektion Kinderchirurgie der Klinik für Allgemein-, Viszeral-, Thorax- und Gefäßchirurgie, Universitätsklinikum Bonn, Bonn, Germany

**Keywords:** Fragility index, Fragility quotient, Meta-analysis, Uninformative statistic, *P* value

## Abstract

**Purpose:**

Meta-analyses occupy the highest level of evidence and thereby guide clinical decision-making. Recently, randomised-controlled trials were evaluated for the robustness of their findings by calculating the fragility index. The fragility index is the number of events that needs to be added to one treatment arm until the statistical significance collapses. We, therefore, aimed to evaluate the robustness of paediatric surgical meta-analyses.

**Methods:**

We searched MEDLINE for paediatric surgical meta-analyses in the last decade. All meta-analyses on a paediatric surgical condition were eligible for analysis if they based their conclusion on a statistically significant meta-analysis.

**Results:**

We screened 303 records and conducted a full-text evaluation of 60 manuscripts. Of them, 39 were included in our analysis that conducted 79 individual meta-analyses with significant results. Median fragility index was 5 (Q25–Q75% 2–11). Median fragility in relation to included patients was 0.77% (Q25–Q75% 0.29–1.87%).

**Conclusion:**

Paediatric surgical meta-analyses are often fragile. In almost 60% of results, the statistical significance depends on less than 1% of the included population. However, as the fragility index is just a transformation of the *P* value, it basically conveys the same information in a different format. It therefore should be avoided.

## Introduction

The fragility index was first described in 2014 by Walsh et al. [1]. It is calculated by an iterative process adding events to the group with a smaller number of events until the *P* value of a two-sided Fisher’s test was at least equal to 0.05. The resulting number of additional events then describes the fragility index. This method has first been described for randomised-controlled trials to assess how fragile, i.e. how many events in the smaller group would be necessary to overturn the trial result [1]. This concept has found wide use in intensive care medicine [2], surgery [3], anaesthesiology [4], and recently in paediatric surgery [5]. However, randomised-controlled trials are scarce in paediatric surgery and often have several methodical shortcomings [6, 7]. Consequently, evidence is often drawn from other study types and their meta-analyses in particular. For them, a variation of the fragility index has been proposed [8]. We used this tool to analyse meta-analyses in paediatric surgery in the decade between 2010 and 2019 to get insight into the fragility of their results.

## Methods

We searched PubMed for meta-analyses on paediatric surgical conditions from 1st January 2010 to 31st December 2019. Eligible records had at least one statistically significant meta-analysis with a dichotomous outcome. The fragility index was calculated by changing the event status in one or more included studies until the pooled effect became non-significant using the online tool [9] provided by Atal et al. [8]. The resulting fragility index was divided by the sample size of the respective meta-analysis to obtain the fragility quotient as described by Ahmed et al. [10], which was then multiplied by 100 to ease interpretation by avoiding very small numbers. These data are presented as medians with interquartile ranges. We also calculated cumulative sums for fragility indices and quotients. We evaluated the association between fragility index and fragility quotient with *P* values by correlation analysis using Pearson’s *R*. Statistical calculations were performed in R (version 3.5.3) with its generic stats4-package [11].

## Results

**Fig. 1:**
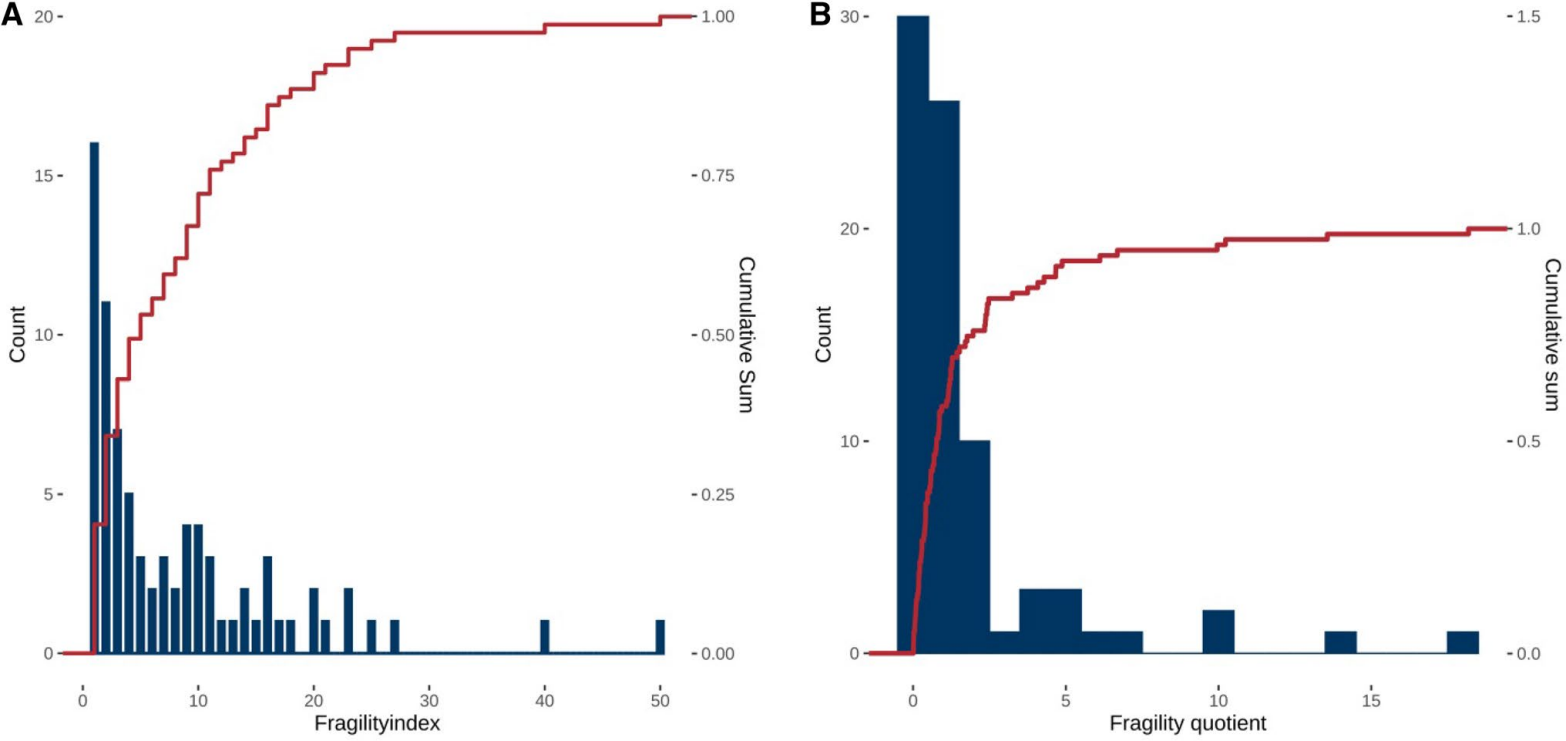
Fragility index and fragility quotient of the included meta-analyses in paediatric surgery. **a** Fragility index of the included meta-analyses with a cumulative sum to better visualise the distribution. **b** Fragility quotient of the included meta-analyses with a cumulative sum to better visualise the distribution. Fragility quotient is displayed as percent to ease interpretation

**Fig. 2:**
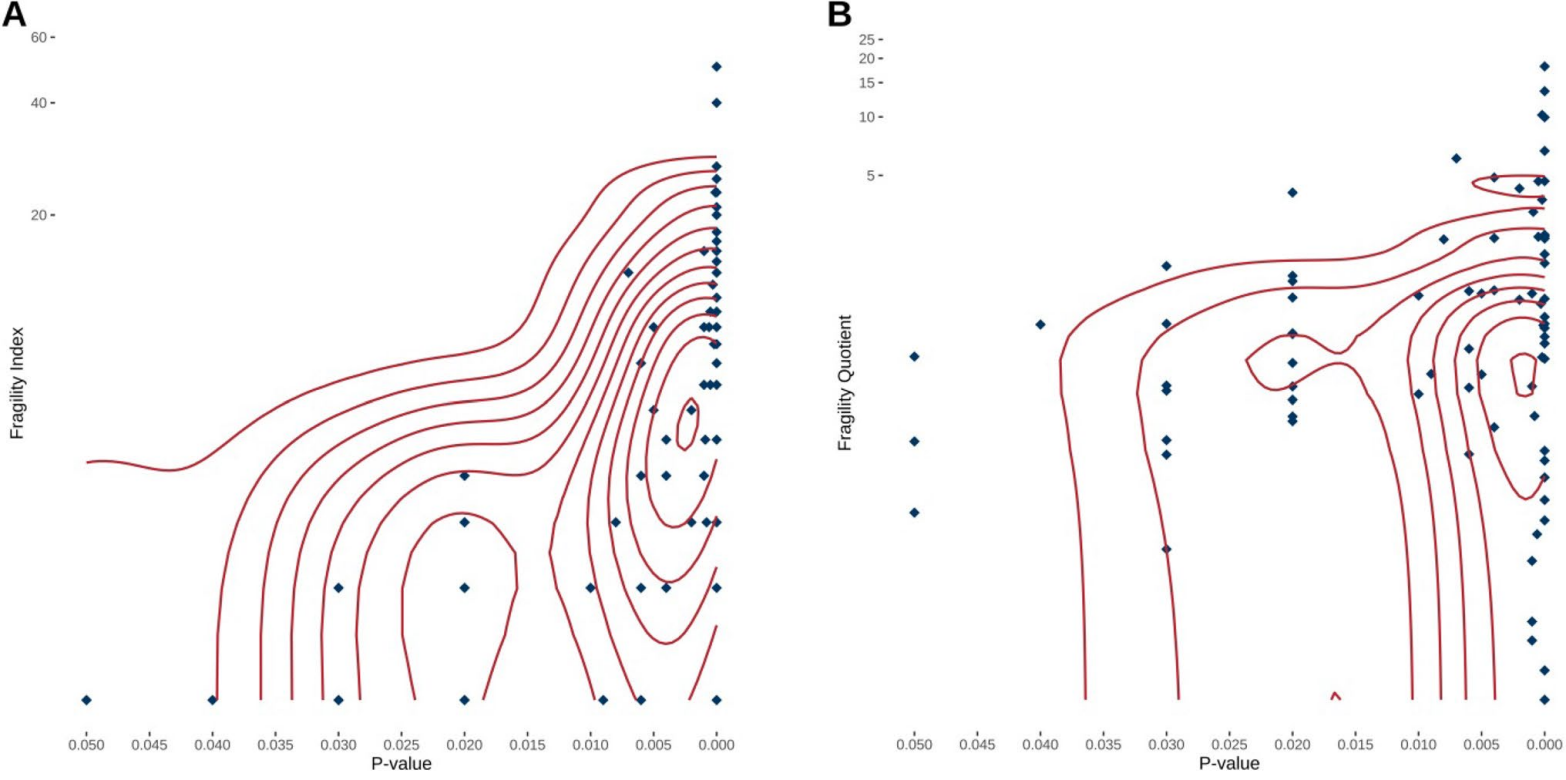
Relationship between fragility index, fragility quotient and *P* values. **a** Relationship between fragility index and *P* values. Fragility index is presented on a logarithmic scale with base two. Diamonds represent individual meta-analyses and lines represent the density. The fragility index is negatively correlated to *P* values with *R* = − 0.49 (95% confidence interval: − 0.3 to − 0.64, *P* < 0.0001). **b** Relationship between fragility quotient and *P* value. Fragility quotient is presented on a logarithmic scale with base two. Diamonds represent individual meta-analyses and lines represent the density. The fragility quotient is negatively correlated to *P* values with *R* = − 0.24 (95% confidence interval: − 0.02 to − 0.44, *P* = 0.0338)

We screened 303 records for eligibility and evaluated 60 full texts. Of them, 39 were included in our analysis with 79 individual meta-analyses that met the inclusion criteria. The median fragility index of included meta-analyses was 5 with an interquartile range from two to eleven in a range from one to 50 (Fig. 1a). The relationship to the total number of included patients was 0.77% at the median with an interquartile range from 0.29% to 1.87% in a range from 0.01 to 18.2% (Fig. 1b). There was a moderate negative correlation between high fragility indices and low *P* values with *R* = − 0.49 (95% confidence interval: − 0.3 to − 0.64, *P* < 0.0001) (Fig. 2a) and low negative correlation between high fragility quotients and low *P* values with *R* = − 0.24 (95% confidence interval: − 0.02 to − 0.44, *P* = 0.0338) (Fig. 2b).

## Discussion

Ideally, meta-analyses represent prime evidence for clinical decision-making, but have been criticised due to an enormous amount of new meta-analyses appearing in the literature every year, many of disputable quality [12]. Correspondingly, many meta-analyses in paediatric surgery have been deemed of low quality based on their so-called AMSTAR-scores (A MeaSurement Tool to Assess systematic Reviews), a checklist score that includes such items as quality of included studies, databases queried, and bias assessment [13]. Conversely, the fragility index has been developed with the intent to give clinicians a tool that enables them to judge how stable a result from a randomised-controlled trial is and how much confidence they can have in the result [1]. Following this first report, many medical specialties examined the results of “their” randomised-controlled trials for fragility and found similar results: trial results are often fragile [2–5]. Consequently, the fragility index has been extended to evaluate meta-analyses [8, 14, 15], and already been expanded to network meta-analyses [16].

We describe the first systematic assessment of meta-analyses in paediatric surgery using the fragility index and quotient. Preceding reports only used this metric for the assessment of their singular meta-analysis and found a fragility index of one [15] and nine [14] for their respective meta-analysis. The fragility index is an absolute metric that does not reflect the sample size of the included studies, for which the fragility quotient has been proposed [10]. However, this metric is calculated from the fragility index and thus similarly problematic.

It has been shown early by simulation studies that the fragility index is influenced by sample size: the larger it is, the higher the fragility index will be [17]. As the fragility index relies on the number of events necessary to render a statistically significant result non-significant [1, 8], it is inherently linked to the *P* value as well. The definition of the *P* value is the probability that the test statistic would have been as large as the observed value given all model assumptions including the test hypothesis were true [18, 19]. In general, a higher *P* value is associated with lower fragility index. For the fragility index, based on Fisher’s test in the analysis of dichotomous outcomes, this translates to two by two tables as extreme as the observed one if all assumptions including the null hypothesis were true. Therefore, decreasing the differences between groups will increase the *P* value and thus decrease the fragility index, because the two by two table becomes more compatible with the null hypothesis. As a consequence, *P* values and fragility indices are highly negatively correlated due to fact that the fragility index is a “repacked” *P *value, or a different presentation of the same notion [17].

The fragility index has, therefore, been described as a “surrogate parameter” [20] for the *P* value. In contrast to it, there is no—although discouraged [21]—dichotomous interpretation of the fragility index [22]. This is even more relevant for the fragility quotient, which is less intuitive than the fragility index [23]. Consequently, the interpretation of both fragility index and quotient is difficult and meaningful values are unknown. Besides the well-described inverse relationship between fragility measures and *P* values [17, 20, 22, 24], the fragility index is linked to sample size; with a fixed number of events in the intervention group, the fragility index varies linearly with the number of events in the control group [24]. Moreover, the fragility index is directly related to larger sample sizes, because larger sample sizes result in smaller *P* values due to the relationship to more extreme tables in two by two tables [17].

This is an aspect that is crucial for paediatric surgery. Studies in surgery are often small due to the smaller target populations and incidences of surgical disease [25], which is even more common in paediatric surgery due to the rarity of congenital anomalies, and further hampered by the rarity of meaningful endpoints in (paediatric) surgery [25]. Penalisation of the smaller study is exemplified by the following comparison of two hypothetical studies: if one smaller study revealed a relative risk reduction of 89%, and this is compared to a larger study with a risk reduction of 20%, they may have equal *P* values of 0.02. In this example, the smaller study would have a fragility index of one compared to a fragility index of nine in the larger study despite a highly different effect size [26]. The fragility index can actively be influenced by the a priori power: the higher this parameter is calculated for, the larger the fragility index will result—in particular if small effect sizes are chased—due to the relationship to sample size [24].

One might argue that this is different for meta-analyses, due to their different method of calculating significant results. This is true in so far as Atal et al. [8] proposed using an iterative process that modified not just one, but as many included studies as necessary to achieve a 95% confidence interval that includes a relative risk of one. However, the basic principle remains the same: a dichotomous assessment of the result depending on its statistical significance. Consequently, the fragility index inherits all problems associated with *P* values and their dichotomous assessment, but without their usefulness [27].

Meta-analyses are difficult to conduct and evaluate [28]. As they get more complex and challenging, we learn more about the process [29] by applying multiple instruments to judge the quality of systematic reviews or meta-analyses, the most prominent among them being the AMSTAR-score [30], the ROBIS-tool [31], and the AMSTAR-2-instrument [32]. The latter two have been found to give similar results [33], whereas the AMSTAR-2-instrument outperformed its predecessor [34], which already identified 75% of all investigated systematic reviews and meta-analyses to be only of poor or fair quality [13]. The original AMSTAR-score has eleven items [30] used to assess the quality of the systematic review by addressing several points that would have a relevant effect on the robustness of findings in the systematic review and meta-analysis. In contrast, the fragility index is only one parameter that is derived from the *P* value of the hypothesis test in the underlying meta-analysis and intended to evaluate the robustness of results. This illustrates that the evaluation of a meta-analysis is much more complex than just calculating a number.

In conclusion, both fragility index and quotient of paediatric surgical meta-analyses are often small, but this finding is of low relevance, because the fragility index is just a permutation of the *P* value. Its calculation cannot replace careful assessment of the included literature and the process used to synthesise the results from them. Therefore, the use of fragility index and fragility quotient needs to be avoided.
